# The Effect of Treatment with Oestradiol Benzoate on Oestrus Expression and Endometrial Oedema in Anovulatory and Cyclic Mares

**DOI:** 10.3390/ani13050938

**Published:** 2023-03-05

**Authors:** Elisa S. M. Silva, John R. Newcombe, Juan Cuervo-Arango

**Affiliations:** 1 Faculty of Veterinary Medicine, Federal University of Uberlandia, Uberlândia 38408-100, Brazil; 2 Warren House Farm, Equine Fertility Clinic, Brownhills WS8 6LU, UK; 3 Equine Fertility Group, Facultad de Veterinaria, Universidad Cardenal Herrera-CEU, CEU Universities, 46115 Valencia, Spain

**Keywords:** mare, oestradiol, endometrial oedema, oestrous behaviour, anoestrus

## Abstract

**Simple Summary:**

Anoestrous mares treated with oestradiol are often used to aid in the collection of stallion semen and as recipient mares to receive embryos when combined with progesterone. The aims of this study were to investigate the dose rate effect of oestradiol benzoate on the duration and persistence of endometrial oedema and oestrous behaviour in anoestrous and cyclic mares. The administration of oestradiol benzoate to seasonally anoestrous mares induced expression of oestrous behaviour and endometrial oedema within 48 h of treatment. Persistence of expression was proportional to the dose and the initial response. A dose of 2 mg was enough to induce oestrous behaviour and endometrial oedema in the majority of anoestrus mares. Regardless of dose, however, there was a strong individual mare effect on the intensity and duration of the response, which was not explained by the weight of the mare. In cyclic mares, treatment with 3 mg of oestradiol benzoate failed to induce endometrial oedema in dioestrous mares; therefore, it can be used as a rapid test to rule out the presence of elevated progesterone concentration in a mare that shows no endometrial oedema despite having a large follicle.

**Abstract:**

Oestrogens treatment is often used to induce oestrus behaviour in anoestrous mares to aid in the collection of stallion semen and as recipient mares to receive embryos when combined with progesterone. However, there are no studies to describe the effect of dose and individual mare on the intensity and duration of the response, in both anoestrous and cyclic mares. In Experiment 1, 13 anoestrous mares were treated with one of five doses of oestradiol benzoate (OB) (1, 1.5, 2, 3 and 4 mg) per mare in five consecutive treatment periods (*n* = 65), to determine the response in terms of endometrial oedema and oestrous behaviour. Experiment 2 and 3 used 3 mg of OB in cyclic mares to confirm or deny the presence of an active corpus luteum (CL). There was a dose rate of OB and individual mare effect (*p* < 0.05) on the intensity and persistence of endometrial oedema and oestrous behaviour. A total of 2 mg OB was enough to induce endometrial oedema and oestrous behaviour within 48 h in most mares. Mares with an active CL did not show endometrial oedema following treatment of 3 mg OB.

## 1. Introduction

The steroid sex hormone 17-β oestradiol is secreted by ovarian follicles and is responsible for oestrous behaviour and other oestrogenic changes in the uterus of mares (i.e., development of endometrial oedema and cervical relaxation) by binding to oestrogen receptors [1]. The oestrous or sexual behaviour in mares depends on the relationship between circulating follicular oestrogens and basal progesterone: it does not begin until peripheral progesterone concentrations have dropped to less than 2 ng/mL, and oestrogen concentrations are beginning to rise [2]. Circulating concentrations of oestrone sulphate increased from basal concentrations (200 pg/mL) 8 days before ovulation to peak concentrations (850 pg/mL) 2 days before ovulation [3]. Furthermore, exogenous treatment with oestradiol in anoestrous mares induced oestrous behaviour within 8 h, whereas daily treatment of progesterone blocked the oestrus-inducing effect of oestradiol [1]. A similar relationship is observed between oestradiol, progesterone and the development of endometrial oedema [4,5]. During low/basal circulating concentrations of progesterone, an ovariectomized mare can show prominent endometrial oedema soon after oestradiol treatment, which can last for as long as 7 days [5], whereas the administration of progesterone 1 day after oestradiol treatment inhibits the development of endometrial oedema. It was concluded that the threshold of circulating progesterone concentration necessary to inhibit endometrial oedema falls between 1 and 2 ng/mL [4].

As a result, the ultrasonographic evidence of endometrial oedema in mares is often used as a clinical sign to confirm that the mare is in oestrus [6]. Most mares follow a common endometrial oedema pattern during oestrus: gradual increase in the oedema score from luteolysis until 2 to 3 days before ovulation, and a rapid decrease thereafter, so that the endometrial oedema at the time of ovulation is returned to the basal score [7]. However, on some occasions a mare may ovulate without showing endometrial oedema and oestrous behaviour in the previous days, despite having basal levels of progesterone and elevated concentration of oestradiol [1,8]. This is termed as silent oestrus or covert oestrus and is more likely to happen after induction of luteolysis with exogenous prostaglandin, with a reported incidence of 6% [8]. Pregnancy rates of mares which did not show endometrial oedema during silent oestrus were reduced (36.4%) [9].

In field conditions, exogenous oestradiol is used mainly to induce oestrous behaviour in dummy (ovariectomized) mares to facilitate semen collection from stallions, and to prepare anoestrous recipient mares to receive embryos following the administration of long-acting progesterone [10,11,12,13]. For those purposes, esterified oestrogens are preferred over the naturally occurring oestradiol-17β, as they are more potent and have longer half-lives [14,15,16].

The objectives of this study were (1) to clinically assess the effect of difference dose rates of oestradiol benzoate on the degree and persistence of oestrous behaviour and on endometrial oedema patterns in individual mares when administered in anoestrus; and (2) to determine whether a single dose of oestradiol benzoate administered to cyclic mares can differentiate between the presence of an active or non-active corpus luteum in the ovary in field conditions. We hypothesized that there would be a dose rate effect of oestradiol benzoate on the magnitude of endometrial oedema and oestrous behaviour in anovulatory mares, and that a single administration of 3 mg oestradiol benzoate would induce endometrial oedema within 24 h of treatment in cyclic mares with no active corpus luteum (CL). 

## 2. Materials and Methods

This experiment was carried out in two locations: (1) the controlled studies (Experiment 1 and 2) were performed in the Faculty of Veterinary Medicine, Uberlandia Federal University, MG, Brazil (18°54′ S); and (2) the field study (Experiment 3) was performed in a private standardbred farm in New South Wales, Australia (33°25′ S). 

### 2.1. Experiment 1 

A total of 13 crossbred mares from the research herd of the Uberlandia Federal University, with a mean age of 12.4 ± 5.9 years (4 to 22 years old) and mean weight of 465 ± 65 kg (350 to 550 kg) were used for two experiments. 

In the first experiment, during the non-breeding season (June to September, southern hemisphere), mares were examined weekly by transrectal ultrasonography to confirm their anovulatory status (absence of a CL, lack of endometrial oedema, and follicles of <20 mm in diameter for a period of at least two consecutive weeks). Once in anoestrus, each mare received 1 of 5 different doses of oestradiol benzoate (1 mg/mL, Sincrodiol, Ourofino, Brazil) administered subcutaneously (1, 1.5, 2, 3 and 4 mg per mare), with a period of rest of one week between treatment periods, so that each mare had received all 5 different doses by the end of the study. The order of the oestradiol benzoate was chosen randomly for each of the 13 mares. Overall, 65 treatment periods were analysed (13 mares multiplied by 5 treatments of different doses) in a crossover design.

**Figure 1:**
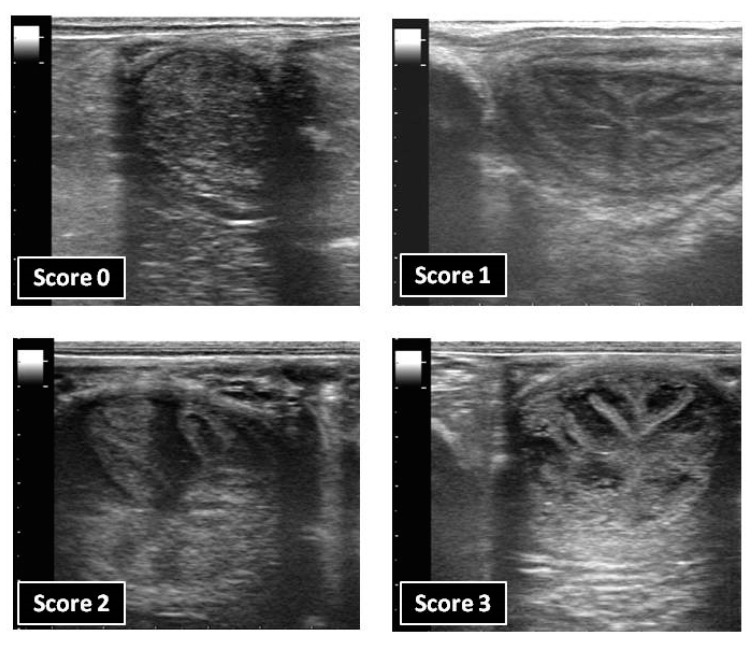
Representative B-mode ultrasonograms of the endometrial oedema scoring system.

After each treatment, the uterus of the mares was examined daily by transrectal ultrasonography with a scanner Mindray DP-6600 vet (Mindray Brasil, Sao Paulo, Brazil), equipped with a linear array transducer of 7 MHz, to determine the endometrial oedema score for a total of 7 days or until the endometrial oedema had returned to basal. The presence of the endometrial oedema was scored subjectively by the same operator on a scale of 0 (minimum degree of endometrial folding) to 3 (maximum degree of endometrial folding) (Figure 1), as described previously [9].

During the same time periods (before treatment and every 24 h after treatment, and for a maximum of 7 days or until the score had return to 0), each mare was presented to the same familiar active teaser stallion in a head-to-head alignment to evaluate the mare’s oestrous behaviour. Initially a distance of at least one metre was maintained between muzzles before the mare was allowed to approach to make contact. This position was maintained for at least one minute and the mare’s behaviour observed. The degree of oestrous behaviour was scored according to the following criteria as described previously [17]: 

Grade 3: oestrous behaviour, approached the stallion willingly and within one minute, with only head to head contact, widened the stance of the hind legs, flexed the hocks and with tail raised, everted the clitoris (‘showing’), and passed small or large quantities of urine.

Grade 2: oestrus, mares behaved as Grade 3 except the position for urination was not adopted until the mare was either allowed to swing her hindquarters towards the stallion or was turned so that her flank and genitalia could be nuzzled.

Grade 1: oestrus, mares approached the stallion positively and allowed nuzzling of their flank and genitalia. Although they continued to evert the clitoris, they did not adopt the urination position within the two-minute teasing period. Most positive signs of oestrous behaviour were shown except hock flexion and urination.

Grade 0.5: mares in anoestrus, and often those coming into or going out of oestrus, usually exhibited a combination of positive and negative signs. Eversion of the clitoris did not occur unless accompanied by a definite negative or aggressive attitude, often accompanied by angry tail swishing. This behaviour is often referred to as ‘showing in spite’. Mares were often reluctant to approach the stallion until forced or approached willingly as if in oestrus to then turn round and kick out. Others, particularly when in anoestrus, would stand passively whilst the stallion nuzzled and bit the flanks without showing any positive or negative signs. Others would stand and ‘show’ initially before kicking out. If the mare showed sufficient positive signs that it was considered that she could be mated, providing a twitch or other restraint such as leg strap was used, the behaviour was classified as Grade 0.5.

Grade 0: no oestrus. Mares which were not in oestrous, exhibited some, but not necessarily all of the following behavioural characteristics: Reluctance to even approach the stallion; desire to swing hindquarters towards the stallion and at least buck if not kick out at him; tail held either tightly down or swished ‘angrily’ from side to side; squirting small quantities of urine apparently in anger rather than the static hock flexion position characteristic of Grade 2 or 3 oestrus; striking out at stallion with front legs on approach; squealing.

### 2.2. Experiment 2

The same mares (*n* = 13) and location as in Experiment 1 were used for this experiment, once the mares entered the breeding season (following the first ovulation after the anovulatory season), between the months of November and February (southern hemisphere). Ovulation was diagnosed by daily ultrasonography as the disappearance of the pre-ovulatory sized follicle. The Day of ovulation was regarded as Day 0. Once a mare had ovulated, she was allocated randomly into one of three experimental groups according to the stage of dioestrus and Day of ovulation: a) early dioestrus (Day 1; *n* = 5), mid dioestrus (Day 6, *n* = 4) and late dioestrus (Day 12, *n* = 4); Each mare received a single treatment of 3 mg of oestradiol benzoate subcutaneously once. The Day of treatment depended on the experimental group (Day 1 for early dioestrus, Day 6 for mid dioestrus and Day 12 for late dioestrus groups, respectively) Before treatment, each mare was scanned by transrectal ultrasonography and the presence of a recent ovulation or a CL and lack of obvious endometrial oedema (score < 1) was confirmed. Furthermore, a heparinized blood sample was taken from the jugular vein once in each mare to determine serum progesterone concentrations immediately before oestradiol benzoate treatment. Mares were scanned as in Experiment 1 for 3 consecutive days after treatment to record the endometrial oedema score at each examination, using the same endometrial oedema score system. 

### 2.3. Experiment 3

During the breeding season of 2020 to 2021 of the southern hemisphere in Australia, standardbred brood mares presented for routine breeding examinations from a commercial stud farm were used in this study. The farm bred 217 mares and had 5 standing stallions on site during that breeding season. The mean age of the mares from this farm was 10.7 years old (3 to 22). Mares were scanned with an ultrasound scanner (Sonosite Nanomaxx, Sonosite Australia, Melbourne, Australia) equipped with a 7–10 MHz linear probe with a colour-flow doppler and B modes available. The routine breeding management included daily scans in every mare intended for breeding. Foaling mares were scanned daily from 5 days after foaling to diagnose the foal heat ovulation which was not used for breeding. Following foal heat ovulation, mares were administered 250 µg cloprostenol (Estrumate 250 µg/mL, MSD Animal Health, Macquarie Park, NSW, Australia) 6 days after ovulation, and then scanned daily from 3 days after cloprostenol treatment until breeding and once ovulation was confirmed. Pregnancy diagnosis was performed 14 days after ovulation. Mares with a negative pregnancy diagnosis were allowed to return spontaneously to oestrus and were scanned daily from Day 17 after ovulation. 

Barren or maiden mares brought for first time to the stud farm were examined by transrectal ultrasonography to diagnose the stage of the oestrous cycle. Mares showing endometrial oedema and follicles >25 mm were scanned daily until breeding and ovulation. Mares with the presence of mature CL, classified according to the luteal echogenicity described previously [18] were administered 250 µg of cloprostenol, and scanned daily from 3 days after treatment, as in foaling mares. 

The breeding management of the farm was as follows: semen was available 3 times a week (Monday, Wednesday, and Friday). Mares in oestrus with endometrial oedema (oedema score ≥ 1), relaxed cervix and a follicle of 28 mm or larger (the optimal pre-ovulatory size follicle for breeding of each mare was based on previous breeding records of each individual mare) were submitted for induction of ovulation and treated with 1.25 mg of deslorelin (Deslorelin Dechra 1.25 mg/mL, Dechra Veterinary Products, Somersby, NSW, Australia), the day before insemination. Mares were scanned the Day of insemination and 24 h after to confirm ovulation and evaluate the post-breeding endometrial reaction to semen. Mares with free-intrauterine fluid were administered 20 IU oxytocin (Syntocyn 20 IU/mL, Troy Animal Healthcare, Glendenning, Australia) intravenously. Mares that did not ovulate within 48 h of insemination were re-inseminated the next day when semen was available. 

Mares showing no endometrial oedema despite the presence of a large pre-ovulatory follicle (>35 mm in diameter), which had been treated with cloprostenol or following a negative pregnancy diagnosis, were examined thoroughly, searching the ovaries for the presence of an active CL. An active and visible CL was defined as the presence of an ultrasonographically detectable CL of 20 mm or larger in diameter with the expected echogenicity according to the age of the CL as described previously [18]. A colour-doppler mode was used to evaluate the CL when there were doubts to distinguish between a regressing CL and an active CL, especially in mares that had been treated with cloprostenol. An active CL showed colour-doppler signal, while a non-active CL (regressing CL or corpus albicans) did not. 

The inclusion criteria for this experiment were: resident mares presented for AI to one of the stallions housed on site, in which the previous Day of ovulation was known (from daily ultrasound examination to diagnose the Day of ovulation) or known interval from the previous treatment with cloprostenol, and presence of a pre-ovulatory sized follicle (>35 mm in diameter) but absence of endometrial oedema. Furthermore, no visible active CL was observed on the ultrasound examination of the ovaries. These mares had been either mares treated with cloprostenol, or scanned 3 days after a negative pregnancy diagnosis. Mares meeting the criteria were treated with a single dose of 3 mg of oestradiol benzoate subcutaneously and scanned within 48 h of treatment to evaluate the presence or absence of endometrial oedema. Mares showing a positive response to oestradiol benzoate treatment were induced to ovulate and inseminated as described previously. 

### 2.4. Hormonal Assay

Blood samples from Experiment 2 were taken from the jugular vein in heparinized 10 mL vacutainer tubes and centrifuged at 2000 G for 10 min. Aliquots of plasma were frozen for later analysis. Progesterone concentrations in plasma samples were measured in duplicate using a commercial ELISA assay (DRG Instruments, Marburg, Germany). Dilution curves generated from a pool of equine plasma with a known high concentration of progesterone and the 0 ng/mL standard supplied with the kit, recommended for serial dilution by the manufacturer, were parallel to the curve produced with the standards supplied with the kit. The intra- and inter-assay coefficient variations (CV) were 6.4% and 6.6%, respectively. The minimal detectable concentration of the assay was 0.16 ng/mL, calculated by adding two standard deviations to the mean optical density value of 10 zero standard replicates and determining the corresponding concentration of progesterone from the standard curve. Cross reactivities were reported as 0.35% for Pregnenolone, 0.3% for 17alpha OH Progesterone, 1.1% for 11-Desoxycorticosterone and 0.2% for corticosterone.

### 2.5. Statistical Analyses

The overall effect of oestradiol benzoate dose on endometrial oedema score and oestrous behaviour was analysed using a general linear model (GLM) of variance (one model for each variable) with a repeated statement to account for autocorrelation between sequential observations of same mares. Sequential data not normally distributed were ranked (oestrous behaviour and endometrial oedema scores) before computing in the GLM (Systat13, Palo Alto, CA, USA). If an effect of group (dose of oestradiol benzoate) or an interaction of dose and Day was significant in Experiment 1, data were examined further by non-parametric Mann–Whitney test within each Day. Furthermore, the effects of dose, weight of mare, and Mare ID on the (a) Day of peak score, (b) Maximum score, and (c) last Day of score 1, for both endometrial oedema and oestrous behaviour scores, were tested on separate GLMs. Spearman ranked correlation was used to determine the level of association between the endometrial oedema score and the grade of oestrous behaviour expressed by the mare on Days 1 and 2 after treatment (days with maximum scores). In Experiments 2 and 3, data are given in a descriptive way: percentage of mares showing endometrial oedema after treatment.

## 3. Results

### 3.1. Experiment 1

**Figure 2:**
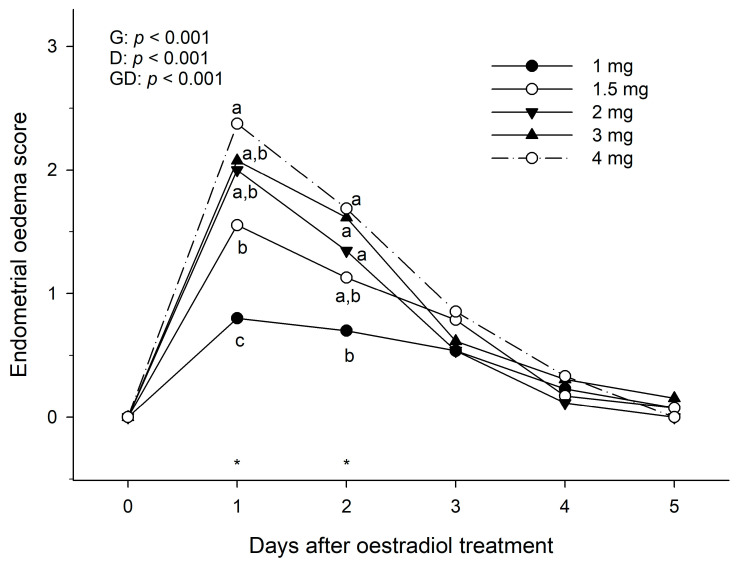
Mean endometrial oedema score in anoestrous mares treated with different dose rates of oestradiol benzoate in a single administration (Day 0). Probabilities for main effects of dose group (G), Day (D), and the group-by-day interaction (GD) are shown. Within a given Day (*), different letters (a, b, c) indicate a significant difference (*p* < 0.05) in endometrial oedema score between dose groups.

**Figure 3:**
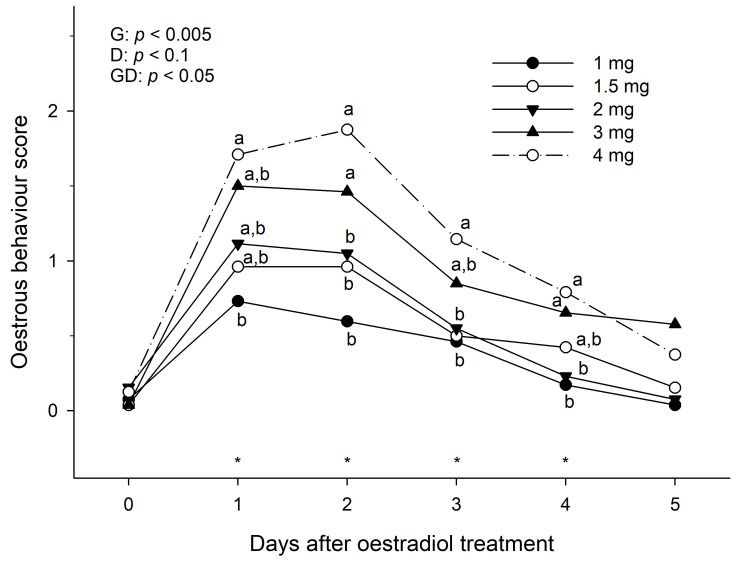
Mean oestrous behaviour score in anoestrous mares treated with different dose rates of oestradiol benzoate in a single administration (Day 0). Probabilities for main effects of dose group (G), Day (D), and the group-by-day interaction (GD) are shown. Within a given Day (*), different letters (a, b) indicate a significant difference (*p* < 0.05) in oestrous behaviour score between dose groups.

The dose rate effects of oestradiol benzoate on endometrial oedema and oestrous behaviour scores in anoestrous mares are depicted in Figure 2 and Figure 3, respectively. For endometrial oedema, there was a significant (*p* < 0.001) dose effect on Day 1 and Day 2 after treatment but was not significant from Day 3 onwards. The maximum dose (4 mg) gave the highest endometrial oedema score (Figure 2). However, this was not different from 3 and 2 mg on Day 1, and not different either to 1.5 mg on Day 2. By Day 4, all mares failed to show some oedema at some of the dose rates. The highest score seen on Day 4 was two at 3.0 mg. By Day 5 of treatment, all mares had return to basal oedema scores, except one mare that showed oedema score 1 after a treatment of 3 mg. The maximum endometrial oedema score, regardless of dose, was obtained 24 h after treatment, which then decreased significantly (*p* < 0.01) by the next 24 h (2 days after treatment) and continued to decrease up to 4 days after treatment (Figure 2).

The effect of dose on oestrous behaviour score was significant during the first 4 days following treatment (Figure 3). The highest doses (3 and 4 mg) of oestradiol benzoate induced a higher (*p* < 0.05) grade of oestrous behaviour on Day 2 after treatment than the lowest doses (2, 1.5 and 1 mg). By Day 5 after treatment, in 15% of cycles (10/65), some mares showed a positive teasing behaviour (Grades 1 and 2), regardless of dose (except for the lowest dose, in which all mares had return to basal score by Day 4). 

The correlation between endometrial oedema score and oestrous behaviour on Day 1 and 2 after treatment was weak (r = 0.33) but significant (*p* < 0.05). For example, at the extremes, a mare could have an endometrial oedema score of three and non-receptive or low-grade oestrous behaviour (Grades 0 to 1), and vice versa: a Grade 3 oestrous behaviour and lower endometrial oedema score (score of 1 or 1.5). However, no mare with a Grade 2 or 3 of oestrous behaviour showed lower endometrial oedema than a score of 1. 

There was a significant (*p* < 0.05) individual mare effect on oestrous behaviour and endometrial oedema score regardless of dose (Table 1). This strong individual mare effect was more marked on the behaviour response to teasing: on the day of treatment (Day 0), three individual mares, on 7 out of 12 occasions, showed weakly positive signs of oestrus. On Day 1, following 1 mg oestradiol benzoate injection, all but 4 mares of the 13 mares showed signs of oestrus which varied in intensity from Grade 0.5 to Grade 2. The number of mares not showing oestrus at any dose rate on any of Days 1, 2, 3, 4 and 5 were 4, 3, 9, 11 and 12 mares, respectively. However, the number of mares showing oestrus following at least one dose rate on Days 1 to 5 was 13, 13, 12, 12 and 8, respectively. One mare never showed oestrus of intensity greater than 0.5 at any dose rate from 1–4 mg while another only showed oestrous intensity of more than 0.5 at the 4 mg dose, and then only for 2 days. By contrast 10 individual mares were still in oestrus (grade 0.5 or more) on Day 5 following doses of 1.5 mg (*n* = 2), 2 mg (*n* = 3), 3 mg (*n* = 6), and 4 mg (*n* = 2). No effect of body weight on mean behaviour scores was found (*p* > 0.1; Table 1).

The individual mare effect on endometrial oedema was obvious in such that a total of three mares did not show any endometrial oedema on Day 1 or 2 after treatments with 1.5 mg (*n* = 2) and 2 mg (*n* = 1). Seven mares failed to show endometrial oedema after treatment of 1 mg of oestradiol benzoate at any of the 5 days after treatment. 

### 3.2. Experiment 2

The mean progesterone concentration (±S.E.M.) of mares in early, mid, and late dioestrus just before oestradiol benzoate treatment was 1.1 ± 0.2 ng/mL, 11.3 ± 0.9 ng/mL, and 10.7 ± 1.1 ng/mL, respectively. None of treated mares in dioestrus showed any endometrial oedema score within 3 days of treatment (zero endometrial oedema score). Some mares from the group of early dioestrus (Day 1 after ovulation) had slight endometrial oedema at the time of oestradiol benzoate treatment which had returned to basal within 24 h, despite oestradiol treatment. 

### 3.3. Experiment 3

There were 36 mares that met the criteria of having a pre-ovulatory sized follicle without signs of an active CL and no endometrial oedema. These cycles represented 10.2% of all oestrous cycles followed during the breeding season in the farm (36/354). Of those 36 mares, 17 (47.2%) had been given cloprostenol (PGF2α) to induce oestrus 3 to 9 days previously. Another 17 (46.2%) mares were 16 to 21 days after ovulation (following a negative pregnancy diagnosis). The last 2 mares (5.5%) were foaling mares which have not had the foal heat ovulation yet. These 2 mares were treated with oestradiol benzoate 25 and 43 days after foaling. The mean follicular diameter and other reproductive parameters of these mares are shown in Table 2.

Following the single administration of 3 mg oestradiol benzoate, 23 mares (63.9%) showed obvious endometrial oedema within 48 h of treatment. The remaining mares (*n* = 13) did not increase the endometrial oedema score following treatment which remained zero. A total of 9 of these 13 mares were from the prostaglandin group. All 36 mares ovulated between 24 and 120 h after oestradiol benzoate treatment. Only mares which showed an endometrial oedema score ≥ 1 after treatment were inseminated. The pregnancy rate 14 days after ovulation was 47.8% (11/23). The overall first cycle pregnancy rate in this stud farm was 54% (191/354).

## 4. Discussion

The hypothesis that the magnitude of endometrial oedema score and oestrous behaviour in anovulatory mares would be oestradiol benzoate dose-dependent is substantiated by the results of this study, as larger doses of oestradiol induced a higher score of endometrial oedema and oestrous behaviour which was maintained for longer than lower doses in anoestrous mares. In addition, in cyclic mares, the treatment of oestradiol benzoate only induced obvious endometrial oedema in mares with no active CL. In terms of induction of endometrial oedema, a dose of 2 mg of oestradiol benzoate was as efficient as the largest dose used in the study (4 mg) to induce a maximum endometrial oedema score within 24 h of treatment. For oestrous behaviour induction, however, it appeared that 3 or 4 mg doses were more efficient in inducing a greater grade of positive oestrous behaviour in anoestrous mares.

It is interesting that such a small dose (2–3 mg) can elicit an obvious endometrial oedema and oestrous behaviour responses for up to 48 h after treatment. This contrasts with previous studies in which a double dose of 10 mg dose of oestradiol benzoate two days apart was recommended to induce oestrous behaviour and increase circulating levels of oestradiol to 15–20 pg/mL in anovulatory mares [19]. According to the results of this study, a dose of 2–3 mg every 2 to 3 days appears to be sufficient to maintain an anovulatory mare in oestrus, to be used, for example, as a dummy mare to aid in the collection of stallion semen [20]. This positive response in terms of endometrial oedema to only 2 mg of oestradiol benzoate agrees with a previous study [21] in which 2.5 mg administration of oestradiol benzoate to anovulatory mares induced a similar uterine oedema pattern and plasma circulating concentrations of oestradiol to those observed in cyclic mares. Therefore, doses of 2 to 3 mg appear to be the quantity of oestradiol benzoate that better mimics the oestrous behaviour and endometrial oedema patterns observed in cyclic mares. 

Since no mare from Experiment 1 showed endometrial oedema or positive oestrous behaviour by 6 days after treatment (even with the highest dose), a gap of 7 days between treatments appeared to be enough rest to evaluate accurately the response to the subsequent treatments. 

The results observed in this study (Experiment 2) following the administration of oestradiol benzoate to cyclic mares (in dioestrus) are consistent with the data showed in the studies from Pelehach and co-workers [4,5] and confirms the failure of oestradiol benzoate to induce endometrial oedema when the mare has elevated progesterone concentrations from an active CL. Similarly, these results agree with a previous study in which the administration of oestradiol followed by progesterone treatment to anoestrous mares did not result in the development of positive oestrous behaviour [22]. In field conditions, especially in stud farms located remotely, where laboratory facilities to measure progesterone are not available, the use of oestradiol benzoate (i.e., 2 to 3 mg) appears a good method to confirm or rule out that the mare is in dioestrus, so that the clinician can make a rapid judgment on whether to inseminate or not a mare, based on the positive (development of endometrial oedema) or negative response. It is not uncommon, however, that following PGF2α administration, some mares respond to the treatment with a partial luteolysis, especially when the PGF2α is given in early dioestrus [23,24]. The CL of mares with partial luteolysis is either not obvious, appears regressed or is no longer visible on ultrasound, but it continues producing varying quantities of progesterone sufficient to prevent the development of endometrial oedema despite the growth of a dominant follicle to pre-ovulatory size [23]. In fact, in Experiment 3, the percentage of mares that did not show endometrial oedema after oestradiol treatment was highest in the group that had received PGF2α to induce oestrus. This may account for a greater number of mares with partial luteolysis which may explain the lack of response to oestradiol benzoate treatment. 

The reason to use this approach (administration of oestradiol benzoate to mares with a large follicle and no endometrial oedema) in a clinical setting (Experiment 3) was double: (1) to determine whether the mare had still some active luteal tissue (which was not diagnosed on ultrasound); and (2) to increase endometrial oedema score before breeding in an attempt to improve the likelihood of obtaining a viable pregnancy, as several studies have shown a positive effect of the duration of endometrial oedema prior to ovulation on subsequent pregnancy rates [9,25,26,27,28].

Unfortunately, in Experiment 3 there was no control group of mares inseminated but that failed to show endometrial oedema, as these mares were not bred for fear of inducing post-mating endometritis [29,30,31], so that meaningful comparisons on fertility of these mares cannot be made. However, an interesting finding is that approximately half of mares treated with oestradiol benzoate soon before ovulation became pregnant, so the treatment did not apparently impair fertilization and embryo development. In a physiological situation in the mare, the levels of circulating oestradiol begin to decline 2 days before ovulation, so that they are low at the time of oocyte release and fertilization [32].

The main limitation of the study is that Experiment 3 was performed in field conditions, with privately-owned mares, so that additional data could not be obtained (i.e., circulating levels of progesterone and oestradiol). 

## 5. Conclusions

In conclusion, a 2 mg dose of oestradiol benzoate in oil given by subcutaneous injection elicits positive oestrus behaviour and obvious endometrial oedema by 24 and 48 h in the majority of mares. Persistence of expression is proportional to the dose and the initial response. Oestrus and endometrial oedema responses may be different due to an individual mare effect, but not weight effect. Maximal oedema response is seen at 24 h but not in all mares at dose rates of less than 3 mg. Mares with an active CL do not show endometrial oedema following treatment of 3 mg of oestradiol benzoate. 

## Figures and Tables

**Table 1:** Effects of dose, mare and weight on endometrial oedema and oestrous behaviour scores in anoestrous mares administered a single treatment of oestradiol benzoate.

Variable	Parameter	Significance (*p* Value)		
		Dose Effect	Mare Effect	Weight Effect
Day of peak score	Endometrial oedema	NS	<0.05	NS
	Oestrous behaviour	NS	<0.01	NS
Last day of score 1	Endometrial oedema	<0.1	<0.05	NS
	Oestrous behaviour	<0.05	NS	NS
Maximum score	Endometrial oedema	<0.001	NS	NS
	Oestrous behaviour	<0.01	<0.05	NS

**Table 2:** Reproductive parameters of mares from Experiment 3 treated with 3 mg oestradiol benzoate due to lack of endometrial oedema despite of having a large follicle and no CL.

Type of Cycle	Mean ± S.E.M. Interval Cloprostenol to Oestradiol (Days)	Mean ± S.E.M. Follicular Diameter at Oestradiol Treatment	Mares with Endometrial Oedema within 48 h (score ≥ 1)	Positive Pregnancy Diagnosis
PGF2α-induced (*n* = 17)	6.1 ± 0.7	38.7 ± 1.1	8/17 (47.1%)	3/8 (37.5%)
Spontaneous (*n* = 17)	NA	40.9 ± 0.9	13/17 (70.6%)	7/13 (53.8%)
Post-partum (*n* = 2)	NA	40.5 ± 0.5	2/2 (100%)	1/2 (50%)

## Data Availability

Not applicable.
